# The Epidemiology of Lead Toxicity in Adults: Measuring Dose and Consideration of Other Methodologic Issues

**DOI:** 10.1289/ehp.9783

**Published:** 2006-12-22

**Authors:** Howard Hu, Regina Shih, Stephen Rothenberg, Brian S. Schwartz

**Affiliations:** 1 Department of Environmental Health, Harvard School of Public Health, Boston, Massachusetts, USA; 2 Channing Laboratory, Department of Medicine, Brigham and Women’s Hospital, Harvard Medical School, Boston, Massachusetts, USA; 3 Department of Environmental Health Sciences, University of Michigan School of Public Health, Ann Arbor, Michigan, USA; 4 Division of Epidemiology, Statistics, and Prevention Research, National Institute of Child Health and Human Development, National Institutes of Health, Department of Health and Human Services, Rockville, Maryland, USA; 5 Departamento de Ecología Humana, Centro de Investigación y de Estudios Avanzados del I.P.N., Unidad de Mérida, Mérida, Yucatán, México; 6 Centro de Investigación de Salud Poblacional, Instituto Nacional de Salud Pública, Cuernavaca, Morelos, México; 7 Departments of Environmental Health Sciences and Epidemiology, Bloomberg School of Public Health and; 8 Department of Medicine, Johns Hopkins School of Medicine, Baltimore, Maryland, USA

**Keywords:** adults, biomarkers, epidemiologic methods, epidemiology, lead, toxicity

## Abstract

We review several issues of broad relevance to the interpretation of epidemiologic evidence concerning the toxicity of lead in adults, particularly regarding cognitive function and the cardiovascular system, which are the subjects of two systematic reviews that are also part of this mini-monograph. Chief among the recent developments in methodologic advances has been the refinement of concepts and methods for measuring individual lead dose in terms of appreciating distinctions between recent versus cumulative doses and the use of biological markers to measure these parameters in epidemiologic studies of chronic disease. Attention is focused particularly on bone lead levels measured by K-shell X-ray fluorescence as a relatively new biological marker of cumulative dose that has been used in many recent epidemiologic studies to generate insights into lead’s impact on cognition and risk of hypertension, as well as the alternative method of estimating cumulative dose using available repeated measures of blood lead to calculate an individual’s cumulative blood lead index. We review the relevance and interpretation of these lead biomarkers in the context of the toxico-kinetics of lead. In addition, we also discuss methodologic challenges that arise in studies of occupationally and environmentally exposed subjects and those concerning race/ethnicity and socioeconomic status and other important covariates.

In the worlds of environmental health and environmental medicine, lead exposure remains one of the most important problems in terms of prevalence of exposure and public health impact. Despite decades of intensive research, lead toxicity also remains one of the most, if not the most, studied subjects of all within the fields of environmental health and environmental medicine. This reflects the large gaps that continue to exist in our understanding of the full implications of lead exposure on health: how lead exposure may impact on chronic diseases; what mechanisms dictate lead’s health effects; how to predict, monitor, and manage lead toxicity; and what factors may modify lead’s effects.

Epidemiologic studies form the body of research most relevant to anticipating human health effects and developing guidelines for preventing and managing human exposures. In recent years, we have seen great advances in their level of sophistication and design. Such advances allow greater inference in terms of issues critical to prevention and public health, such as potential causality, dose–response relationships, and susceptible subpopulations.

Two of the articles in this mini-monograph are reviews of the effects of lead on the cardiovascular system and upon cognitive function in adults (Navas-Acien et al. 2007; Shih et al. 2007). Issues of methodology determine the confidence to be placed on the validity of the evidence presented in such reviews and the strength of the appropriate inferences. In this present article we attempt to review and address the major methodologic issues common across studies. Chief among the recent developments in methodologic advances has been the refinement of concepts and methods for measuring individual lead dose. This includes appreciating distinctions between recent versus cumulative doses and acute versus chronic effects, as well as the use of biological markers in epidemiologic studies to measure these parameters. In this article, we review this topic in some depth.

We also address important issues related to epidemiologic design, such as cross-sectional versus longitudinal studies; community-based versus occupational studies; and the identification and control of bias and confounding. Our goal in this article is to provide the methodologic basis to interpret the major issues that are of relevance to the articles in the mini-monograph as well as future studies intending to investigate the effect of lead on health outcomes.

## Measurement of Lead Dose

### Concepts and overview

In reviewing studies of the health effects of lead, it is critical to understand the available lead biomarkers in terms of how they represent external exposure (in terms of timing, duration, magnitude, and accumulation), how they are influenced by metabolic factors (organ distribution, compartmental dynamics, the influence of physiologic factors), and how the combination of these considerations impacts inferences regarding the health effects of lead. In this section, our goal is to persuade the reader that the most informative recent epidemiologic studies of the impact of lead on health are those that were able to derive estimates of both recent and cumulative lead dose for each study participant.

To achieve this end with greatest precision and accuracy, such studies have incorporated measurements of lead in both blood (whole blood, using standard chemical assays such as graphite furnace atomic absorption spectroscopy) and bone (using noninvasive *in vivo* K-shell X-ray fluorescence [KXRF] instruments). Blood lead levels are an indicator of circulating lead that captures variation in recent external lead exposure as well as lead that has been mobilized from tissue stores (mostly bone). Lead levels in tibia and patella provide an indication of cumulative dose over decades (particularly cortical tissue in tibia) as well as the largest pool of lead in the body that is available for mobilization into blood. The latter phenomenon is heightened at times of high bone resorption (e.g., during pregnancy, aging, postmenopause). Taken together, blood and bone lead levels have provided recent epidemiologic studies with the best available assessment tools for estimating both recent and cumulative lead. It is also noted that an acceptable surrogate for cumulative lead dose that does not require KXRF measurement of lead in bone can be derived by using repeated measures of blood lead over time of exposure to derive time-integrated indices such as working lifetime time-integrated blood lead levels [IBL—the area under the curve of blood lead vs. time, also termed cumulative blood lead index (CBLI)]. Such measures have been demonstrated to be well-correlated with tibia lead levels (Roels et al. 1995; Somervaille et al. 1988), and this is reviewed in detail below. However, epidemiologic studies (particularly of nonoccupational cohorts) that have had access to valid measures of blood lead repeated over years of exposure have been rare.

Blood lead levels measured in epidemiologic studies with valid instruments and standardized calibration and quality control procedures have been reported in the literature for more than 35 years. Bone lead levels measured by *in vivo* KXRF were begun in some research laboratories in the 1980s, but it was not until the mid-1990s that reports began to emerge of KXRF-measured bone lead levels in relation to potential health indicators from epidemiologic studies with large enough sample sizes (e.g., 100 or more subjects) to have substantial statistical power. Below we discuss these issues in more detail.

### Issues of validity related to recent and cumulative lead dose

When reviewing studies on the health effects of an “exposure,” it is critical to evaluate the validity of biological tests (i.e., biomarkers) used to measure “exposure.” Validity, in this context, incorporates notions of measurement precision and accuracy as well as the value of the metric in predicting the health outcome(s) of interest.

With respect to lead toxicity, biological measurements of lead have been used to measure lead dose for decades. Indeed, the most commonly employed of such tests, the measurement of lead in whole blood, has become one of the few human tests of an environmental toxicant that is widely available in commercial U.S. laboratories and that has been legislated as a test for monitoring exposure required by many states (e.g., lead screening in children) and by the federal government [e.g., the U.S. Occupational Safety and Health Administration (OSHA) lead standard for workers].

However, a recurrent theme of this mini-monograph is that the blood lead test cannot be assumed to be the best and only metric of lead exposure that matters. A blood lead level reflects, for the most part, recent lead exposure (i.e., over weeks to months) from environmental or occupational sources. Although lead in blood is also in equilibrium with bone lead stores, its variability mostly reflects changes in external exposure. Over the past 10 years or so, epidemiologic studies have generated growing and undeniable evidence that the most important standard for predicting some adverse health outcomes is not recent lead exposure; rather, it is cumulative lead exposure that occurs over many years, with or without the additional dimension of latency (i.e., the passage of time that may be needed for a toxic outcome to be expressed).

The concept that cumulative lead exposure may be more important than recent lead exposure derives partly from animal studies demonstrating that the long-term administration of low doses of lead for varying lengths of time result in animals with similar blood lead levels but with levels of end-organ toxicity that are directly proportional to the varying cumulative doses [for recent examples of such studies with respect to kidney and brain toxicity, see Ghorbe et al. (2001) and Lasley and Gilbert (2002), respectively]. The importance of cumulative dose (and latency) has also been supported by longitudinal epidemiologic studies in which, compared with subjects with low lead exposure, individuals with a clear history of high lead exposure in the past have been found to have a higher profile of disease despite having current blood lead levels similar to those without such a history [e.g., rates of hypertension and adverse reproductive outcomes (Hu et al. 1991a, 1991b), respectively]. Although peak dose and timing of the dose may also have been important to the observed health effects in these subjects, evaluation of cumulative dose would not have been possible if only current blood lead levels had been measured.

Perhaps most important, however, has been the development of KXRF instruments for the *in vivo* measurement of lead in bone, which provide a direct biological metric for estimating retained cumulative lead dose in humans (Landrigan and Todd 1994). Over the last 12 years this development has allowed investigators to conduct epidemiologic studies that specifically focus on the potential adverse health outcomes resulting from an estimate of lifetime cumulative dose.

Many of the studies using KXRF, including those reviewed in this mini-monograph concerning the toxicity of lead in adults in the cardiovascular system and on cognitive function, have generated considerable interest in the environmental medicine community. For the researcher, it is as if the adverse health effects of smoking or diabetes had always been limited to considering how many cigarettes per day an individual was currently smoking or what the blood sugar level was today rather than cumulative pack-years of smoking or the hemoglobin A1C level, respectively. Recent exposure, in this case, as reflected by the blood lead level, remains a critically important measurement, but by expanding the tool chest of measurement methods to include measures of cumulative dose, the ability to understand the full expression of lead toxicity has been markedly increased.

### Lead uptake, distribution, metabolism, and excretion

The principal routes of exposure and absorption of lead are through ingestion and inhalation (White et al. 1998). Absorption in the gut is partial, influenced by physical form, chemical species of lead, and the presence of other nutrients and dietary cations such as iron and zinc. The molecular mechanism for lung absorption is unknown, but it is well known that if the physical form is of respirable size (i.e., < 1 μm, such as lead fume generated by burning lead paint), absorption is efficient (> 90%; Rabinowitz et al. 1977). Lead-containing particles > 2.5 μm in diameter are deposited in the ciliated regions of the nasopharyngeal and tracheobronchial airways, where they are passed to the gastrointestinal tract by the mucociliary lift mechanism and then subject to intestinal absorption.

Once absorbed through either ingestion or inhalation, lead enters the bloodstream where it is predominantly bound to erythrocyte proteins (Barltrop and Smith 1972; Bergdahl et al. 1997; Church et al. 1993; O’Flaherty 1993; Rabinowitz 1991; Rabinowitz et al. 1976; Simons 1984, 1988), with an average clearance half-time after a short-term limited exposure of approximately 35 days from whole blood (Rabinowitz 1991). Clearance occurs through distribution into soft tissues and bone as well as excretion, primarily through kidney filtration and elimination in urine. A small amount of lead is also excreted in feces, sweat, hair, and nails.

Lead circulates widely and is found in all organs and tissues; it also crosses the blood–brain barrier and placenta, making the brain and developing fetus among the targets of concern (Hu 1998). On a molecular level, lead binds to many proteins, especially to thiol and carboxyl groups, and mimics calcium in many biologic pathways (Goldstein and Ar 1983; Kern et al. 2000; Rabinowitz 1991; Rabinowitz et al. 1973).

### Biomarkers of lead dose

Blood lead levels, hereafter referred to as “BLLs,” are typically measured in whole-blood specimens that have been collected by venous phlebotomy or by fingerstick (Schlenker et al. 1994) into tubes containing an anticoagulant. To measure lead concentrations in such specimens, most laboratories use graphite furnace atomic absorption spectroscopy, which typically has a limit of detection (LOD) of 1 μg/dL. Some laboratories still use anodic stripping voltammetry, which has an LOD of 5 μg/dL. A direct readout portable instrument (LeadCare Analyzer; ESA Laboratories, Bedford, MA) is also now available that can make measurements of BLLs in the field with a LOD of 1 μg/dL. All such methods have well-standardized quality assurance/quality control procedures that involve the regular use of calibration standards and participation in proficiency protocols.

Although the half-time of lead in blood of 35 days is widely quoted in the literature, it is critical to appreciate that this estimate is a reflection of the 120-day life span of erythrocytes and only applies in practice if the total exposure is short (e.g., < 30 days). If lead exposure is long-term (i.e., with a duration of years), upon cessation the kinetics of clearance of lead from blood is considerably more complicated, with an initial rapid decline in levels reflecting partial clearance from blood and other soft tissues followed by a much slower clearance, reflecting the replenishment of soft-tissue pools of lead with lead from long-lived deposits in bone. Thus, as a biological marker of dose, blood lead levels can be a reflection of acute external exposure, internal bone lead stores released into blood, but, most commonly, a steady-state mixture of both external exposure and internal stores with almost no ability to distinguish between either.

With respect to lead and bone, it has been well established from autopsy studies that the skeleton contains 90–95% of lead burden in adults and 80–95% in children (Barry and Mossman 1970; Hu et al. 1989; Schroeder and Tipton 1968). Roughly 15% of circulating lead per day is incorporated into bone (Rabinowitz et al. 1976), where it substitutes for calcium in the hydroxyapatite of bone mineral during the normal and ongoing process of bone deposition. The bulk of lead in bone is contained within long-lived compartments of cortical (clearance half-time of decades) and trabecular (clearance half-times of years to decades) bone, with comparatively small amounts of lead in bone tissue compartments that rapidly exchange with extracellular fluid and plasma.

The decades-long half-time of lead in cortical bone makes it a dosimeter of retained cumulative lead dose when measured either at autopsy, by biopsy, or by noninvasive *in vivo* techniques such as KXRF. In comparison, the much shorter half-time of lead in trabecular bone makes it somewhat less reliable as a dosimeter of cumulative lead dose, but it identifies trabecular bone as a measure that reflects a large pool of stored lead that may be more bioavailable than cortical bone lead (Hu et al. 1998). A combination of decreased “external” lead exposure and normal rates of bone resorption can result in bone constituting the predominant source of circulating lead in elderly individuals (Hu et al. 1996b) or in individuals with past long-duration high exposure and low current exposure. There is some inconsistency in the literature on this point. For example, in one study of approximately 1,000 subjects 50–70 years of age with mainly environmental lead exposure, the mean blood lead level was approximately 4 μg/dL and the mean tibia lead level was approximately 19 μg/g, but the Pearson’s *r* correlation between the two was only 0.12 at a time of low ongoing environmental lead exposure (Martin et al. 2006; Schafer et al. 2005). This suggests that in persons whose peak lead exposures were decades in the past, the current tibia lead is not very bioavailable and thus does not contribute much to current blood lead levels. Greater levels of circulating lead may derive from bone during physiologic states accompanied by heightened bone resorption, such as pregnancy and lactation, postmenopausal osteoporosis (Korrick et al. 2002; Webber et al. 1995), and hyperthyroidism (Goldman et al. 1994).

When considered in relation to a long-term conceptual model of lead exposure, dose, and the ability to retrospectively estimate dose in individuals, it is clear that bone lead levels provide a cumulative dose metric that is completely distinct from that of blood lead levels, particularly among individuals whose peak lead exposures had occurred in the past (Hu et al. 1998).

Except for rare circumstances, there is little or no value in measuring lead in urine or hair. Because of the pharmacokinetics of lead clearance, urine lead changes more rapidly and may vary independently of BLLs. Moreover, urine lead is less validated than blood lead as a biomarker of external exposure, or as a predictor of health effects. Lead in hair, particularly in working populations, may partially reflect external contamination rather than only internal lead dose. Laboratory analysis for lead in hair is not standardized and is highly variable (Harkins and Susten 2003; Seidel et al. 2001). Lead in plasma or serum (Cake et al. 1996) has recently received attention as a biological marker that is distinct from whole blood lead and that by virtue of representing the fraction of lead in blood that is most biologically available to cross cell membranes such as the placenta may be better at predicting some forms of toxicity than whole blood lead levels (Hu et al. 2006). However, plasma or serum lead levels are extremely low (< 1% of whole blood lead levels); are particularly vulnerable to contamination issues (including contamination from the hemolysis of red cells, where > 99% of lead in whole blood resides); require extremely labor-intensive methods for their collection, processing, quality control, and measurement (Smith et al. 2002); and are likely most relevant to very short-latency, acute health effects.

The literature on the health effects of lead exposure also includes studies that have measured chelatable lead burden. This is measured as lead excreted in urine collected for 4–24 hr after administration of intravenous EDTA or oral administration of dimercaptosuccinic acid (DMSA) (Lee et al. 1995). In a number of small studies, it was suggested that EDTA-chelatable lead could be used as an estimate of current body burden, a metric closely related to cumulative lead dose (Sokas et al. 1988). Other groups have thought of chelatable lead as the bioavailable body burden (Lee et al. 1995). Although chelatable lead has been used in large-scale epidemiologic studies, some groups have concluded that chelatable lead probably does not additionally contribute to causal inference when added to studies that have already measured blood lead and bone lead (Dorsey et al. 2006; Weaver et al. 2005). As such, we do not consider these metrics further in this review.

### Measurement of bone lead using KXRF

Details regarding the measurement of lead in bone using *in vivo* KXRF have been reviewed previously (Chettle et al. 1991; Todd et al. 1992a), as has the use of bone lead in epidemiologic studies (Hu et al. 1989, 1998; Landrigan and Todd 1994). Here we only briefly reiterate key points necessary to interpretation of information in this mini-monograph. As noted above, direct *in vivo* measurements of lead in bone by KXRF have been used in epidemiologic research since the mid-1990s, and their usefulness derives from their ability to provide estimates of cumulative lead dose as well as of a reservoir of lead that may be released into circulation at high levels during periods of heightened bone resorption. This section contains a brief discussion of KXRF methodology for the nonphysicist, focusing on aspects that are of practical value for research and clinical applications. Discussion and critiques of a second technique, LXRF, may be found elsewhere (Rosen and Slatkin 1993).

Most current KXRF instruments use cadmium-109 as the low gamma radiation source to provoke the emission of fluorescent photons from the anatomical target area (Hu et al. 1998). The measurement is automated and typically requires 30 min. The net lead signal is determined after subtraction of Compton background counts, using a nonlinear, least-squares fit program (Chettle et al. 1991). The lead fluorescence signal is then normalized to the elastic or coherently scattered X-ray signal, mainly from calcium present in bone, resulting in units of micrograms of lead per gram of bone mineral. By normalizing the measurement to calcium counts, the measurement is rendered insensitive to variations in bone shape, size, density, histomorphometry, overlying tissue thickness, and a small amount of movement (Somervaille et al. 1985). Radiation dosimetry studies have demonstrated that a typical ^109^Cd KXRF measurement gives extremely low effective dose values of < 0.1 μSv (Todd et al. 1992b), significantly below a proposed limit of negligibility of 10 μSv (Hall 1988). Studies with repeated measurements have indicated the instrument provides a high degree of precision of the point and measurement uncertainty estimates compared with chemical analyses of lead-doped phantoms (Aro et al. 1994) and cadaveric legs (Aro et al. 2000). Regarding the latter, repeated measurements of tibia bone shown by chemical analysis to have lead concentrations in the 20–30 μg/g range (common values for environmentally exposed late middle-age adults) typically demonstrate SDs of around 3–4 μg/g (i.e., coefficients of variation of 15–20%) Studies support the inference that a single-site bone lead measurement is representative of total skeletal burden (Hu et al. 1990; Wittmers et al. 1988). However, several groups have recommended separate measurements of cortical and trabecular bone sites (Chettle et al. 1991) to deal in part with kinetic differences for lead in these compartments (Kim et al. 1997). The utility of measuring at cortical and trabecular sites for causal inference in epidemiologic studies has also been recently questioned (Dorsey et al. 2006; Weaver et al. 2005).

Several caveats need to be noted with respect to factors that can affect KXRF measurement precision. First is that precision is partially dependent on the mass of bone being measured, so measurements in women and children typically have higher measurement uncertainties. Second, thickness of the soft tissues overlying the bone being measured can influence precision, although for midtibia shaft this is generally not a large concern. Precision can be affected by errors in the placement of the instrument in relation to target bone, movement of the subject’s leg out of the field of measurement, or curtailed measurement time. Finally, it is important to note that the quality control and assurance procedures for KXRF instruments typically involve the use of calibration standards and procedures by individual laboratories. National or international standards for the intercalibration of KXRF instruments have not yet been established. A process of creating intercalibration standards, suggested intercalibration protocols, and standard reference material consisting of ground bone is currently under way.

### Bone lead levels: interpretation and implications

In the interpretation of bone lead levels, a few issues must be kept in mind. First, for low concentrations of lead in bone, in epidemiologic studies it is recommended that tibia lead be used as a continuous variable, retaining all values, including those below the LOD and those below zero (Kim et al. 1995). Although there is some debate about the utility of measuring cortical and trabecular sites, we believe that if *a priori* hypotheses are developed that clearly distinguish the purpose of comparing different lead biomarkers, then it is reasonable to explore the contrasting associations. However, it must be noted that as more associations are evaluated the probability of chance associations increases. Third, studies are increasingly identifying how bone density and/or bone resorption rates may influence bone lead measurements (Goldman et al. 1994; Hu 1998; Tsaih et al. 2001), because, as noted, bone lead contains bone mineral concentration in the denominator. To date, no epidemiologic studies that have evaluated associations of bone lead with health outcomes have tried to control for bone mineral density or resorption rate. Finally, it is important to note that measures of cumulative lead dose, such as tibia lead levels, give no information on acute short-term “peaks” or other time-varying aspects of lead exposure which, under certain circumstances, may be important to appreciate (such as documenting episodes of short-term acute clinical lead poisoning).

### Estimating cumulative lead dose with the CBLI

Since KXRF remains available in only approximately seven research institutions in North America, measurement of BLLs will remain the mainstay of biological monitoring for lead-exposed workers for the foreseeable future. To address cumulative dose using the readily available BLLs, an alternative methodology is to use repeated and/or estimated measurements of blood lead over time to calculate a CBLI (Somervaille et al. 1988). CBLI is mathematically equivalent to the area under the curve of BLLs versus time, is approximately equivalent to multiplying the average BLL by the number of years of exposure, providing units of microgram-years per deciliter, and is similar to what other authors have termed the IBL index. If repeated BLLs are available, the trapezoidal rule can be used to compute the area under the curve to arrive at CBLI (Appendix A).

Validation studies comparing CBLI with tibia lead provide evidence that CBLI is an estimate of cumulative lead dose. Eleven separate determinations have been made of slopes of the relation between tibia lead and CBLI (Armstrong et al. 1992; Cake et al. 1996; Erkkila et al. 1992; Hu et al. 1991a; McNeill et al. 2000; Somervaille et al. 1988). Each study also reported sample size and the SE of the slope, which, across the studies, ranged from 0.028 to 0.067. Although we did not perform a formal meta-analysis, pooling of these data and weighting by sample size provides an estimated slope of 0.05 [95% confidence interval (CI), 0.046–0.055]. These data support the notion that CBLI and tibia lead are correlated and the estimated slope of this relation across studies has been remarkably consistent with a narrow CI. In addition, this slope indicates that tibia lead concentration (micrograms per gram) can be estimated as 5% of the CBLI (microgram-years per deciliter). Additional research in this area would be useful to see if the relationship is nonlinear, particularly within subsets of the population, such as the young versus the elderly.

This knowledge allows the magnitudes of effects to be compared across studies that have used the two measures and for equivalences to be estimated in the average BLL over time that would result in a given tibia lead level. For example, using the logistic regression model based on data presented from the Normative Aging Study, an increase of 29 μg/g tibia lead (which was the increase of bone lead from the midpoints of the lowest to highest quintiles) would be associated with a 1.74-times increase in the odds of hypertension regardless of the starting bone lead level (Hu et al. 1996a). This model predicts that a 1.5-times increase in the odds of hypertension would be associated with an increase in tibia lead of 21.3 μg/g, which corresponds approximately to an increased CBLI of 400 μg-years/dL.

A potential limitation with interpreting CBLI and health effects is that many of the studies that have attempted to use CBLI have involved worker cohorts that had much higher BLLs in the past, but lower levels at the time data were collected and the cumulative lead dose (i.e., CBLI) was calculated. Even though not all lead-exposed workers have had required BLL testing over time, it may be possible to extrapolate an approximate CBLI using available BLL results along with a careful occupational history to estimate the intensity and duration of lead exposure. For example, if a worker is exposed at the current OSHA-permissible exposure limit and permitted to have the maximum BLL (40 μg/dL) each year for a working lifetime (40 years), the estimated CBLI would equal 1,600 μg-years/dL, whereas a worker with a BLL of 20 μg/dL each year for 10 years would have a CBLI of about 200 μg-years/dL.

Another point to consider when interpreting CBLI is that some of tibia lead (and blood lead) may derive from higher past environmental (nonwork) exposures. Older workers may have higher baseline bone lead levels from living at times when environmental exposures were higher. The population mean BLL was 13 μg/dL in the late 1970s (Annest et al. 1983; Pirkle et al. 1994). By the late 1990s, the mean BLL had fallen to < 3 μg/dL (Pirkle et al. 1998). This point is relevant to cumulative dose estimation with both tibia lead and CBLI; that is, if associations are observed, we cannot clearly distinguish early from mid-life from later life exposures using these summary metrics and thus must interpret the critical periods of exposure with caution.

Thus, in this mini-monograph, we believe that epidemiologic studies that measured either tibia lead or CBLI have adequately estimated cumulative dose. The consistent relation between tibia lead and CBLI in validation studies clearly supports this conclusion.

## Other Methodologic Considerations

Epidemiologic research has evolved considerably in its thinking about study design and causality inference. Recent studies on lead toxicity have been more rigorous in their consideration of such important issues as selection bias, confounding, effect modification and other forms of interactions, and complex causal pathways. In the following sections we briefly review other issues of relevance to the interpretation of associations in epidemiologic studies that have relevance to the two systematic reviews in this mini-monograph (Navas-Acien et al. 2007; Shih et al. 2007).

### Occupational versus environmental studies

While studies of occupationally exposed populations provided initial data about the harmful effects of lead at high levels of exposure, environmental studies are much less troubled by the healthy worker effect, survivor cohorts, and other sources of bias inherent to occupational studies. Environmental studies also have the capacity to encompass much larger sample sizes with more socioeconomic and racial/ethnic diversity than occupational studies.

These are important differences. In some cases, researchers conducting studies in subjects with high chronic occupational lead exposure have failed to observe the adverse impacts associated with lower levels of lead in environmental settings. For example, several studies of renal function in smelter workers have found no evidence of clinical renal dysfunction or changes in markers of tubular dysfunction compared with controls (Gerhardsson et al. 1992; Omae et al. 1990; Roels et al. 1994; Wang et al. 2002). Similarly, some occupational studies have not found significant differences in cognitive symptoms or psychosocial disturbances between occupationally exposed workers and controls (Parkinson et al. 1986).

There are many potential explanations for null associations in occupational studies. Occupational studies tend to be based on small sample sizes, making them vulnerable to type II error. Some studies had nonexposed, or control, groups of workers with BLLs well above current background-exposure levels (< 5 μg/dL), which is likely to underestimate the effect being studied. Perhaps the most important problem is the vulnerability of occupational studies to the healthy worker effect, that is, the bias inherent in studying populations of workers who remain after the departure of sicker and/or more susceptible workers, especially problematic in cross-sectional studies of current workers. In studies that compare health effects in workers with general population controls, the healthy worker effect could explain why associations may not be observed, because workers are, especially for symptomatic conditions, more likely to be healthier than the general population. This problem is often manifested as an attenuation of exposure–response curves in occupational studies at high exposure levels (Stayner et al. 2003). If lead causes most of its health effects at low levels, occupational studies that examine only the high end of the dose–response range could miss associations at the lower end or across the entire range (Nuyts et al. 1993). It has also been reported that there may be selection bias by δ-aminolevulinic acid dehydratase genotype, as lead exposure and exposure duration increase and symptomatic workers leave the workplace (Schwartz et al. 1995).

Such selection bias can be mitigated in two ways. First, although more difficult, this bias could be avoided in occupational studies that assembled complete cohorts and randomly selected workers for study, including those who left the workplace early or late in their careers. Of interest is that studies that have assembled cohorts of all workers ever employed in a given plant or industry have reported many significant findings not previously observed (Schwartz et al. 2000, 2001, 2005; Stewart et al. 1999). Second, longitudinal studies, in comparing subjects with themselves in changes in health status over time, are somewhat less susceptible to this kind of bias, whether occupational or environment. Community-based studies of the general population avoid the healthy worker effect entirely, making them an especially important study design, although a similar form of bias can be encountered in studies limited to just older subjects in the general population.

In this mini-monograph, both occupational and environmental studies were chosen for inclusion in the systematic reviews (Navas-Acien et al. 2007; Shih et al. 2007). When evaluating the associations of cumulative lead dose with health outcomes, investigators need to acknowledge that nonoccupational sources of lead exposure were present for all members of the general population in the United States, including lead workers, throughout most of the 20th century until public health interventions progressively removed lead from gasoline and many consumer products during the 1970s and 1980s (Agency for Toxic Substances and Disease Registry 1999; Annest et al. 1983; Pirkle et al. 1998). Lead remains a low-level and ubiquitous neurotoxicant in the environment and is found in measurable levels in all individuals (Hoppin et al. 1995). Thus, current tibia lead levels may represent a mix of environmental exposures and potential occupational exposures.

### Race/ethnicity, socioeconomic status, and other factors/covariates

Environmental lead exposure differs by race/ethnicity and socioeconomic status. Persons with low socioeconomic status (e.g., educational attainment, income, assets) have been known to have higher blood lead levels throughout at least the period of the recurrent National Health and Nutrition Examination Survey (NHANES) blood lead surveys (Annest et al. 1983; Elreedy et al. 1999; Pirkle et al. 1998). Several investigators have also reported higher bone lead levels in minorities compared with whites. For example, Martin et al. (2006) recently reported that tibia lead levels among a population-based sample of individuals 50–70 years of age in Baltimore, Maryland, were 30% higher in African Americans than in whites (Martin et al. 2006). Lin et al. (2004) reported higher tibia and patella lead levels in minorities compared with predominantly white subjects who were older than 55 years and living in Boston, Massachusetts (Lin et al. 2004). One study reported higher tibia and patella lead levels in blue-collar workers compared with white-collar workers and this association was modified by race/ethnicity (Elmarsafawy et al. 2002).

The strong associations of cumulative lead dose with race/ethnicity and socioeconomic status raises methodologic concerns. Factors that in the past were simply termed “confounding variables” are now more carefully evaluated as potential mediators (i.e., in the biological causal pathway), moderators (i.e., risk modifiers), direct causes, or otherwise parts of complex causal pathways (Kraemer et al. 2001). It is now understood that such complex causal pathways also apply to lead exposure and chronic disease, including cognitive dysfunction, hypertension, and renal dysfunction. These pathways can include connections between individual-level indicators (e.g., age, sex, race/ethnicity, socioeconomic status); behavioral risk factors; biological factors (e.g., genetics); social factors (e.g., social capital, social cohesion); lead dose (i.e., both recent and cumulative); health conditions (e.g., diabetes, heart disease, hypertension); and other biological markers predictive of disease (e.g., homocysteine levels) that may be thought of as either outcomes by themselves or as intermediate pathological states that result in other conditions (e.g., renal dysfunction, cognitive declines).

What are the implications of the fact that race/ethnicity and socioeconomic status may be causally related to cumulative lead dose? Although low socioeconomic status is associated with higher BLLs in population-based surveys, early life lead exposure has been shown to cause intellectual impairment and worse educational outcomes (Banks et al. 1997; Canfield et al. 2003; Needleman et al. 1979, 1996, 2002; Pocock et al. 1994), which in turn may influence socioeconomic status attainment in later life. Thus, although early studies of lead and cognitive function argued that controlling for education was an important necessity, when including the contribution of early life lead exposures, the potential reciprocal causation may lead to an underestimation of the association between lead dose and cognitive function if education is included in regression models. This has also been recently discussed in the context of lead and blood pressure (Martin et al. 2006).

A similar issue has been raised concerning race/ethnicity. To the extent that race/ethnicity serves as a proxy for other factors influenced by early lead exposure and also adversely affect cognitive function or cardiovascular outcomes (Navas-Acien et al. 2007; Shih et al. 2007), adjusting for race/ethnicity could underestimate the overall effect of that early lead exposure. Some authors have thus argued that there should not be adjustment for race/ethnicity (Martin et al. 2006; Shih et al. 2006). If later life lead exposure also affects the measured cognitive or cardiovascular outcome, then to the extent that race/ethnicity also serves as a proxy for factors that influence later life lead exposure and these outcomes, not adjusting for race/ethnicity may introduce bias.

Thus, it can be concluded that inclusion of race/ethnicity in models evaluating relations of cumulative lead dose and cognitive function or cardiovascular outcomes could lead to an underestimation of the direct effect of lead. Given these complex causal pathways, we believe relations of tibia lead and these outcomes are likely to be best estimated by parsimonious regression models that control for such variables as age, sex, and testing technician, for example, but not necessarily those that include race/ethnicity and socioeconomic status, which is at odds with what has been concluded by other authors (Goodman et al. 2002; Lindgren et al. 1996) and with our earlier thinking on this issue (Balbus-Kornfeld et al. 1995). It may be most informative in the future if analyses were explicitly reported (and appropriately interpreted) that both included and excluded race/ethnicity and measures of socioeconomic status.

The ideal solution to such a conundrum, although not often possible, would be to have separate measures of early-life and late-life lead exposures and/or direct measures of the underlying factors for which race/ethnicity is serving as a proxy (LaVeist 1994) and possibly applying statistical methods such as marginal structural models to account for variables that are both mediators in the pathway to exposures and confounders of later exposures (Robins et al. 2000). Another issue of concern when considering race/ethnicity is that there might be plausible physiologic differences by race/ethnicity that affect the association between lead and cognitive or cardiovascular outcomes. In such a case, one must consider effect modification by race/ethnicity in analyses, although not without the same considerations raised above regarding the precise factors for which race/ethnicity is serving as a proxy (LaVeist 1994). Although stratified analysis by race/ethnicity is one approach to these concerns, very few epidemiologic studies are designed to be adequately powered for stratified analysis, and thus loss of statistical significance is often the result. Stratified analysis should not be necessary if evaluation of effect modification in a single model (i.e., by inclusion of an interaction term between lead dose and race/ethnicity) reveals no evidence that the association with lead dose differs by race/ethnicity.

A number of important individual-level factors, in addition to race/ethnicity and socioeconomic status, are frequently considered in the body of literature on the health effects of lead. When comparing associations of health outcomes with blood and bone biomarkers, it is essential to recognize that factors such as age, sex, and elevated bone turnover accompanying osteoporosis (Silbergeld et al. 1988; Webber et al. 1995) may modify lead toxicokinetics. These factors may co-vary with age, race/ethnicity, and sex, and genetic polymorphisms, complicating consideration of these issues.

An increasing body of evidence suggests that lead is associated with a number of health conditions that are also causes of cognitive decline, including hypertension (Hertz-Picciotto and Croft 1993; Hu et al. 1996a; Nash et al. 2003; Sharp et al. 1987), elevated homocysteine levels (Schafer et al. 2005), and psychiatric symptoms (Rhodes et al. 2003; Schwartz et al. 2001). Each of these health conditions may mediate or moderate (or confound, in the absence of temporality) relations between lead dose and cognitive function.

Two important health behaviors—tobacco and alcohol consumption—have been linked with risk of cardiovascular and cognitive outcomes. This begs the question of whether it is critical to adjust for tobacco and alcohol consumption in evaluating relations of lead dose with cardiovascular and cognitive outcomes. The numerous studies of tobacco and alcohol consumption and its relations with cognitive function are conflicting (Carmelli et al. 1999; Cervilla et al. 2000; Crawford et al. 2001; Elias et al. 1999; Elwood et al. 1999; Hendrie et al. 1996; Schinka et al. 2002, 2003). We do not believe that control for these variables is absolutely necessary and can be guided by modeling and appropriate interpretation of causal pathways.

As another example, smoking is often included in models of hypertension, and its impact on blood pressure remains an important potential mechanism for its status as a risk factor for end-organ dysfunction (Orth 2004). Tobacco is well known to have been contaminated by lead arsenate pesticide in the past and smoking has been identified as a risk factor for increased cumulative lead burden (Hu et al. 1996b). Thus, it is possible that epidemiologic studies of hypertension that adjust for smoking are “over-controlling” for a risk factor (smoking) that is associated with the exposure of interest (lead), underestimating the direct effect of lead. Similar concerns pertain to alcohol, which has been linked to the pathogenesis of hypertension [with moderate to heavy consumption (Beilin and Puddey 2006)] and is also well-known risk factor for elevated lead exposure (Lee et al. 2005).

## Conclusions

Two articles in this mini-monograph (Navas-Acien et al. 2007; Shih et al. 2007) are systematic reviews of studies that have examined associations of lead dose with cognitive and with cardiovascular outcomes. For the reasons reviewed in this article, both reviews focus on studies that measured both recent dose, mainly with blood lead, and cumulative dose, mainly with tibia lead, but also with trabecular bone lead and CBLI. By comparing and contrasting associations of blood lead with those using a cumulative dose metric, studies can attempt to distinguish the acute effects of recent dose from the chronic effects of cumulative dose. Although, for the reasons reviewed above, comparison of associations with these different biomarkers does not allow absolute differentiation of these different effects, studies that have compared associations can more readily generate hypotheses about how lead influences health, which dose period is more important, and whether health effects are more likely to be reversible or irreversible.

## Appendix A. Calculation of Cumulative Blood Lead Index

CBLI gives a useful summary of a set of sequential blood lead measurements from individuals. For example, how could the following data from an individual followed over time from 1996 be summarized?

**Table:**

No.	Date	Blood lead (μg/dL)
1.	1 June 1997	6.0
2.	1 June 2001	1.0
3.	1 June 2004	1.0

To calculate the CBLI using the Trapezoidal Rule, take a piece of graph paper and draw a set of axes, the horizontal axis representing time, the vertical axis representing blood lead concentration. Label the first time point after the origin on the horizontal axis with the date of the first available blood lead test. Label the vertical blood lead axis with the range of expected blood concentration. Draw a dot representing the coordinates (time and lead concentration) of the three measurements. Connect the dots with straight lines.

Draw a horizontal line across the plot at blood lead = 0. From each dot draw a vertical line to the zero blood lead line. Draw horizontal lines connecting each vertical line so that a triangle and a rectangle are formed between each adjacent pair of data points.

Measure the height in units of lead and the width in units of time of each triangle and rectangle in the plot and record them on the appropriate parts of the triangles and rectangles.

Use the formula for area of a triangle and area of a rectangle to calculate the area of each triangle and rectangle drawn:

The first time interval in the data set, 1 June 1997 to 1 June 2001, is 4 years. The sum of the heights of the stacked triangle and rectangle (trapezoid) of the first data point is 6 μg/dL, with the height of the triangle being 5 μg/dL and the height of the rectangle below it 1 μg/dL. The area of the triangle of the first interval is (5 × 4)/2 = 10 μg-years/dL; the area of the rectangle below it is 4 × 1 = 4 μg-years/dL. Write each area inside the triangle or rectangle.

The sum of the area of the triangle and the rectangle gives the total CBLI of the patient between 1997 and 2001 (10 + 4 = 14 μg-years/dL).

Move on to the next time interval, 1 June 2001 to 1 June 2004. Repeat the drawing of the triangle and the rectangle. In this interval the blood lead concentration remained the same, so draw only a rectangle. The height of this rectangle is 1 μg/dL and the length is 3 years. The area of this rectangle is 1 × 3 = 3 μg-years/dL, which is also the value of the CBLI for the 3-year interval. Write the area inside the rectangle.

To calculate the total CBLI, add the CBLI values previously calculated as areas. In this example, the CBLI for the first interval was 14 μg-years/dL and for the second interval was 3 μg-years/dL, so the total CBLI for the interval between 1 June 1997 and 1 June 2004 is 17μg-years/dL.

The Trapezoidal Rule method of calculating CBLI will slightly overestimate CBLI for subjects with suddenly decreased exposure and will slightly underestimate CBLI for subjects with lead exposure that is seasonal or occasional, depending on the blood sampling interval. Better accuracy is assured by shorter sampling intervals, at least every 6 months. Longer sampling intervals may be used for subjects with unchanging exposure, although with some loss of accuracy.
